# The effect of acupuncture-related therapies in animal model of postmenopausal osteoporosis: a meta-analysis and data mining approach

**DOI:** 10.3389/fendo.2025.1617154

**Published:** 2025-10-15

**Authors:** Guangbin Yu, YingYing Gao, Hongyuan Song, Guizhen Chen, Yunxiang Xu

**Affiliations:** ^1^ Shenzhen Bao’an Traditional Chinese Medicine Hospital, Guangzhou University of Chinese Medicine, Shenzhen, Guangdong, China; ^2^ Clinical Medical School of Acupuncture, Moxibustion and Rehabilitation, Guangzhou University of Chinese Medicine, Guangzhou, Guangdong, China

**Keywords:** acupuncture, postmenopausal osteoporosis, meta-analysis, data mining, acupoint

## Abstract

**Purpose:**

This meta-analysis and data mining aimed to investigate the effectiveness of Acupuncture-Related Therapies for animal models with Postmenopausal Osteoporosis (POMP) and to summarize the acupoints involved.

**Methods:**

This systematic review was conducted a comprehensive search of animal experiments using acupuncture-related therapies for the treatment of PMOP up to April 1, 2025. The primary outcome was bone mineral density (BMD). The secondary outcome indicators were estradiol(E_2_), blood calcium, osteocalcin(OC) and alkaline phosphatas(ALP).Meta-analysis was used to evaluate its efficacy, and data mining was used to explore the protocol for acupoint selection.

**Results:**

27 Animal Experiments encompassing 548 animals with PMOP were analyzed. Meta-analysis displayed that compared with the conventional drug group, the acupuncture-related therapy group significantly increased the Estradiol (mean difference [MD] 2.37,95% confidence interval [95%CI] 1.15 to 3.58), Femoral BMD (mean difference [MD] 1.25,95% confidence interval [95%CI] 0.65 to 1.87), lumbar BMD (mean difference [MD] 1.88,95% confidence interval [95%CI] 1.27 to 2.49), Tibia BMD (mean difference [MD] 1.63,95% confidence interval [95%CI] 0.56 to 2.69). Data mining revealed that Zusanli (ST36), Shenshu (BL23), Guanyuan (CV4), and Sanyinjiao (SP6) were the core acupoints for PMOP treated by Acupuncture-Related Therapies.

**Conclusion:**

Acupuncture improved BMD and estrogen levels in animal models of PMOP. ST36, BL23, CV4, and SP6 are the core acupoints for acupuncture in PMOP, and this program is expected to become a supplementary treatment for PMOP.

## 1 Introduction

Postmenopausal osteoporosis (PMOP) is a metabolic bone disorder characterized by low bone mass and altered bone microarchitecture, which increases the risk of fractures. It typically occurs in women within 5 to 10 years after menopause (1). According to the World Health Organization (WHO) criteria, osteoporosis is defined as a bone mineral density (BMD) that is 2.5 standard deviations or more below the average value for young, healthy women, corresponding to a T-score of less than -2.5 SD (2). Clinically, osteoporosis is associated with pain—particularly in the spinal region—and, in severe cases, may lead to spinal deformities and fragility fractures. Additionally, PMOP causes a significant decrease in BMD and estradiol levels, while increasing osteocalcin, alkaline phosphatase, and blood calcium levels.

The prevalence of postmenopausal osteoporosis varies by country. For instance, researchers estimate that 10.2 million adults over the age of 50 in the United States have osteoporosis, while in Canada, more than 2 million individuals are affected by the condition (3).

Beyond its high prevalence, osteoporosis also imposes substantial economic costs on countries worldwide. For example, recent data from China indicate that the median per-admission inpatient cost for osteoporotic fractures is ¥18,587. In Mexico, the direct cost associated with more than 75,000 fragility fractures was estimated at $256.2 million in 2010, with a projected increase of 41.7% expected by 2020 (4).

Postmenopausal osteoporosis is currently treated using various therapeutic methods, with conventional therapy primarily consisting of calcium (5), vitamin D (6), and estrogen (7). Although drugs such as denosumab and bisphosphonates are used to treat postmenopausal osteoporosis, the risk of fractures and decreased bone density increases once patients discontinue these medications (8). Research indicates that the antiresorptive effects are rapidly reversed within 9 to 30 months after discontinuation (9). The high economic costs and severe side effects of these drugs are often intolerable for patients with postmenopausal osteoporosis. Therefore, novel therapeutic strategies need to be explored to improve bone mineral density and reduce fracture risk in patients with postmenopausal osteoporosis.

Acupuncture originated from traditional Chinese medicine and has been shown to be effective in treating a wide range of diseases (10, 11). According to the World Health Organization, acupuncture literally means puncturing with a needle (12). However, it may also involve the application of other types of stimulation to specific points (13). All methods that use electricity, pressure, heat, or other means to stimulate specific acupoints to achieve therapeutic effects—including traditional acupuncture—are generally referred to as acupuncture-related therapies (14). Therefore, the specific types of acupuncture therapies discussed in this manuscript include manual acupuncture, electroacupuncture, ear (auricular) acupuncture, auricular pressure, acupoint catgut embedding, and acupressure. Acupuncture-related therapies also encompass forms combined with moxibustion or medication, such as warm needling, fire needling, and acupoint injection (15).

The efficacy of acupuncture in the treatment of postmenopausal osteoporosis (PMOP) has been extensively studied. In Asia, numerous clinical studies and animal experiments have systematically validated the effectiveness of acupuncture for PMOP, providing a solid foundation for its clinical application. Meanwhile, a clinical trial conducted in Europe demonstrated that acupuncture can effectively alleviate pain symptoms in PMOP patients, improve their quality of life, and has significant sustained effects (16). A recent study conducted in Africa also showed that acupuncture not only relieves PMOP-related pain but also significantly improves bone density in patients (17). These research findings consistently indicate that acupuncture has a stable therapeutic effect in enhancing bone health, relieving pain, and improving quality of life in PMOP patients. Its therapeutic value has been verified across different regions, including Asia, Europe, and Africa, fully supporting the global promotion and application of acupuncture as a reliable non-pharmacological intervention.

In recent years, numerous studies have demonstrated that acupuncture can significantly improve clinical symptoms associated with postmenopausal osteoporosis (PMOP) (18). However, fragmented evidence limits its clinical application. Animal models of PMOP are essential for testing acupuncture-related therapies. For clinicians and researchers specializing in postmenopausal osteoporosis, studying the effects of acupuncture on animal models can provide a deeper understanding of the physiological and pathological mechanisms underlying these therapies, thereby enhancing their ability to apply them effectively in patient care (19–21). To more accurately simulate the physiological condition of postmenopausal women, PMOP models are typically generated by performing ovariectomy on animals. Ovariectomy is a surgical procedure that removes the ovaries, ceasing their function and causing a sudden drop in estrogen levels. This process mimics the estrogen decline that occurs after menopause in humans and is used to study changes in bone metabolism and the efficacy of various treatments. First, this model rapidly induces a sharp decline in estrogen levels, accurately simulating the core pathophysiological mechanism of PMOP (22, 23). Second, its bone loss characteristics closely resemble those in humans, with changes in cortical and trabecular bone and microstructural damage consistent with human patterns (22–25). Additionally, the bone metabolic mechanisms are comparable, both resulting from estrogen deficiency that leads to impaired bone turnover, characterized by increased bone resorption and reduced osteoblast function (22–25). Furthermore, the response to treatment is consistent; interventions such as estrogen replacement and bisphosphonates effectively reverse bone loss, mirroring human treatment outcomes (22–25). Finally, this model is practical, efficient to establish, easy to operate, and cost-effective, making it suitable for large-scale studies (23, 24).

Meta-analysis and data mining of animal experiments are now recognized as effective methods for enhancing the value of animal studies in guiding clinical research (26). This paper evaluates the efficacy of acupuncture-related therapies in PMOP model animals through meta-analysis and data mining, aiming to further explore the potential of these therapies for improving PMOP in clinical settings.

## 2 Method

### 2.1 Meta-analysis methods

#### 2.1.1 Protocol and registration

Our study was conducted based on the checklist of the preferred reporting items for systematic reviews and meta-analyses (PRISMA) guidelines 2020 (27)(S1 File). This study was registered with PROSPERO (registration number: CRD420251012487).

#### 2.1.2 Literature search

China National Knowledge Infrastructure Database (CNKI), China Biomedical Literature, China Science and Technology Journal Database, Wanfang Database, Embase, PubMed, the Cochrane Library, and Web of Science were searched of clinical studies on Acupuncture-Related Therapies for postmenopausal osteoporosis up to April 1,2025. An example of the PubMed search strategy is shown in **Supplementary Figure S1**.

#### 2.1.3 Inclusion and exclusion criteria

##### 2.1.3.1 Inclusion criteria

①Subject: Controlled study on the effects of acupuncture-related therapies on a postmenopausal osteoporosis animal model. ② Intervention: Acupuncture-related therapies were used as the intervention. ③ Control: Conventional treatment group. ④ Outcome Indicators: The primary outcome indicator was bone mineral density (BMD), and the secondary outcome indicators were estradiol (E_2_), blood calcium, osteocalcin, and alkaline phosphatase (ALP). ⑤ Type of Study: Controlled animal experimental research, with no restrictions on the modeling method, species, or strains of the experimental animals, and no language restrictions on the included literature.

##### 2.1.3.2 Exclusion criteria

①Duplicate entries of titles and duplicate publications;②reviews, meta-analyses, and *in vitro* studies; ③studies for which full text and complete data were not available;④ studies of low quality.

##### 2.1.3.3 Literature screening, data statistics, and risk of bias

First, the retrieved primary literature was imported into EndNote, and the included studies were selected through a stepwise screening process based on predefined inclusion and exclusion criteria. Second, the baseline information from each study was recorded in a table of basic characteristics. The extracted data included the author, year of publication, country where the study was conducted, study period, and total number of animals included in the study. Finally, the risk of bias was assessed using the SYRCLE (Systematic Review Centre for Laboratory Animal Experimentation) risk-of-bias assessment tool (28). All tasks were performed independently by Yingying Gao and Hongyuan Song, with any disagreements resolved by Gangbin Yu.

##### 2.1.3.4 Statistical analysis

Meta-analysis was performed using Stata 17.0 (STATA.MP; StataCorp LP, College Station, TX, USA) and R 4.4.2. Since all outcome indicators were continuous variables, the standardized mean difference (SMD) was used as the effect size statistic, and its 95% confidence interval (95% CI) was calculated. A p-value of less than 0.05 was considered statistically significant. Statistical heterogeneity was assessed using the I² statistic. When heterogeneity was low (I² ≤ 50%), a fixed-effects model was applied for the meta-analysis; when heterogeneity was high (I² > 50%), a random-effects model was used. If more than 10 studies were included, funnel plots were generated to evaluate publication bias.

### 2.2 Data mining methods

#### 2.2.1 Literature search

China National Knowledge Infrastructure Database (CNKI), China Biomedical Literature, China Science and Technology Journal Database, Wanfang Database, Embase, PubMed, the Cochrane Library, and Web of Science were searched of clinical studies on Acupuncture-Related Therapies for postmenopausal osteoporosis up to April 1,2025. An example of the PubMed search strategy is shown in **Supplementary Figure S1**.

#### 2.2.2 Inclusion and exclusion criteria

The inclusion criteria were: (i) Studies reporting the use of acupuncture-related treatments as the main intervention were included;(ii) Whether or not a randomized controlled method was adopted, they all met the inclusion criteria;(iii) The research subjects were postmenopausal osteoporosis animal models. (iv) the experimental group received acupuncture-related treatments, and the control group was a conventional treatment group; (v) The acupoint records were complete; (vi) The efficacy of acupuncture was definite.

The exclusion criteria were: (i) Studies with inconsistent research content or inconsistent primary outcome measures; (ii) Studies with a large difference in sample size between the treatment group and the control group and inconsistent baseline levels; (iii) Dissertation studies and studies with a large number of sample dropouts; (iv) Poor quality studies, such as those that don’t clearly describe the type of animals they used; (v) For repeatedly published or data-overlapping literatures, only one was retained; (vi) Data not available; (vii) Studies in which the intervention measures of the treatment group were non-acupoint therapies such as abdominal acupuncture and scalp acupuncture.

#### 2.2.3 Standardization of acupoint names

The names of acupoints included in the studies were standardized based on the “Names and Locations of Commonly Used Acupoints in Laboratory Animals”. For example, “Guanyuan” was standardized as “CV4”, and “Zigong” was standardized as “EX-CA1”.

#### 2.2.4 Data analysis

A database was created using Excel 2021 to record the frequency of acupoint usage. SPSS Modeler 18.0 was employed to perform frequency and association rule analyses. The association rule analysis was conducted using the Apriori algorithm with the following settings: support ≥ 20%, confidence ≥ 75%, and lift ≥ 1.0, to identify the core acupoint combinations used in acupuncture-related therapies for PMOP animal models. Support measures the frequency of an itemset appearing in the dataset. Confidence represents the likelihood of a subsequent itemset occurring given a set of prerequisite items. Lift is used to determine whether a rule has practical application value. Based on this parameter, the association rule analysis identifies core acupoint combinations with practical significance, consisting of acupoints that frequently occur and are closely connected. The acupoints comprising these core combinations are defined as core acupoints. Cytoscape 3.10.0 was used to generate complex network diagrams. SPSS Statistics 26.0 was utilized to conduct a systematic cluster analysis of acupoints and to create an iceberg plot and a dendrogram from the cluster analysis.

## 3 Result

### 3.1 Meta-analysis

#### 3.1.1 Literature screening

**Figure 1:**
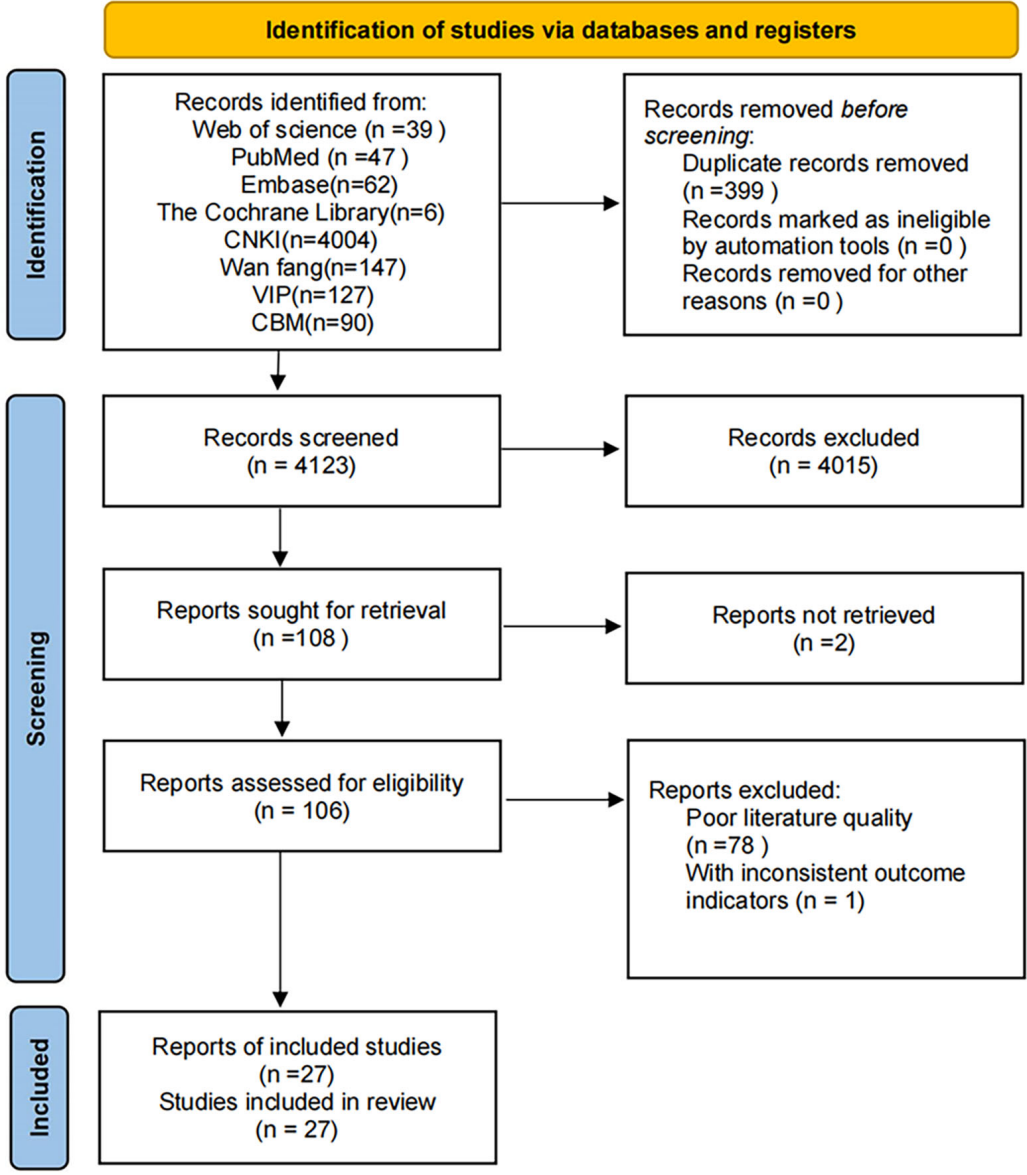
Study flow diagram.

The search yielded 4,535 relevant studies, of which 27 were included after systematic screening (29–55) (**Figure 1**).

#### 3.1.2 Basic characteristics of included studies

The 27 included studies (29–55), all conducted at centers in China and Japan, involved a total of 548 animals. Of these, 275 received conventional treatment, while 273 received acupuncture-related therapies combined with conventional treatment (**Table 1**).

**Table 1:** Basic characteristics of the included studies.

Author Name	Research Center	Sample Size (E/C)	Acupuncture Treatment	Acupoints	Treatment Duration	Outcome
Wenping Zhang 2007 (29)	China	8/8	acupuncture	Sanyinjiao(SP6), Neiguan (PC6)	24weeks, once a week	①②③
Na Shi 2022 (30)	China	8/8	acupoint catgut embedding	Shenshu(BL23), Zusanli(ST36)	12weeks, once a week	①②
Atsushi Takeda 2018 (31)	Japan	10/12	acupuncture	Shenshu (BL23)	12weeks, 3 times a week	①②
Xiao-Sheng Lin 2019 (32)	China	15/15	acupoint application therapy	Shenque(CV8)	12 week, once a day	①③
Hong-Du Wang 2015 (33)	China	9/10	electroacupuncture	Jingmen(GB25), Huan tiao(GB30), Yangling quan(GB34), Xuanzho ng(GB39)	90 days, once a day	①③
Jing He 2013 (34)	China	7/7	electroacupuncture	Zusanli(ST36), Pishu(BL20), Shenshu(BL23)	14 days, once a day	①②
Jun Zhou 2012 (35)	China	10/10	electroacupuncture	Zusanli(ST36), Sanyinjiao(SP6)	12 week, once a day	①
YiFeng 2015 (36)	China	10/9	electroacupuncture	Xuanzhong(GB39), Mingmen (GV4)	4 week, once a day	①②③
HUAILING FAN1 2016 (37)	China	10/10	electroacupuncture	Guanyuan(CV4)	1 month, once a day	①③
Xuefeng Zheng 2018 (38)	China	10/10	electroacupuncture	Pishu(BL20), Shenshu (BL23)	90 day, once a day	①③
Pan Fangfang 2011 (39)	China	11/12	electroacupuncture	Shenshu(BL23),Ming men(GV4),Xuanzhong(GB39),Guanyuan(CV4),Zigong(EX-CA 1),Zusanli(ST36),San yinjiao(SP6)	90 day, once a day	①②
Shi Na 2022 (40)	China	8/8	electroacupuncture	Guanyuan(CV4), San yinjiao(SP6), Shenshu(BL23), Zusanli(ST36)	12 week, once a day	①②
Sun Guanghua 2018 (41)	China	8/8	electroacupuncture	Sanyinjiao(SP6), Yang lingquan(GB34), Yinli ngquan(SP9)、 Zusanli(ST36)	12 week, once a day	②③
Bao Shengyong 2012 (42)	China	12/12	electroacupuncture	Guanyuan(CV4), Zusa nli(ST36), Shenshu(B L23), Geshu(BL17), D azhu (BL11)	12 week, once a day	②③④
Li Pei 2014 (43)	China	6/6	electroacupuncture	Mingmen (GV4)	20 day、once a day	②
Jin Xiaoqing 2003 (44)	China	8/7	electroacupuncture	Extra point-B2(Lumbar 1)(EX-B2(L1)),Extra point-B2(Lumbar2)(E X-B2(L2)),Extra point-B2(Lumbar 3)(EX-B2(L3)),Extra point-B2(Lumbar 4)(EX-B2(L4)),Huant iao(GB30),Zusanli(S T36),Shenshu(BL23) Pishu(BL20),Mingme n (GV4)	12 week, three a week	①②③
Qu Yuanyuan 2024 (45)	China	20/20	electroacupuncture	Guanyuan(CV4), Zusanli(ST36)	8 week, once a day	①②
Zheng Xiaohua 2024 (46)	China	8/8	electroacupuncture	Mingmen (GV4), Huantiao(GB30)	12 week, once a day	①③
Yuan Chunchun 2020 (47)	China	8/9	acupuncture	Shenshu(BL23), Pishu(BL20), Zusanli(ST36), Dazhui(GV14)	30 day, once a day	①②
Liu Baoxin 2019 (48)	China	10/10	acupoint catgut embedding	Shenshu(BL23), Jingmen(GB25)	12 week, twice a month	①②③④
Bai Dengyan 2021 (49)	China	15/15	electroacupuncture	Shenshu(BL23), Sanyi njiao(SP6), Guanyuan(CV4), Zusanli(ST36)	12 week, once a day	①③
Chen Jing 2022 (50)	China	12/12	acupoint catgut embedding	Shenshu(BL23), Jingmen(GB25)	3 week, once every ten days	①③④
Zhang Chuqiong 2022 (51)	China	8/8	electroacupuncture	Guanyuan(CV4), San yinjiao(SP6), Shenshu(BL23), Zusanli(ST36)	12 week, five times a week	①
Zhang Guangmei 2020 (52)	China	8/8	acupuncture	Shenshu(BL23), Xuanzhong(GB39)	60 day,Once a day, then stop for two days after ten days	②
Li Li 2019 (53)	China	10/9	acupuncture	Shenshu(BL23), Zusanli(ST36)	90 day,Once a day, then stop for five days after ten days	①
Guo Di 2023 (54)	China	12/12	acupuncture	Zusanli(ST36), Shens hu(BL23), Guanyuan(CV4)	8 week,once a day	①④
Qian Kang 2018 (55)	China	12/12	Zuogui Pill+ Moxibustion	Changqiang(GV1)	90 day,once a day	①

①Bone mineral density (including lumbar spine, femur, tibia, etc.), ②blood biochemistry (serum estradiol, calcium), ③bone turnover markers (osteoprotegerin, osteocalcin) and ④alkaline phosphatas are the outcome indicators.

#### 3.1.3 Risk of bias assessment

**Figure 2:**
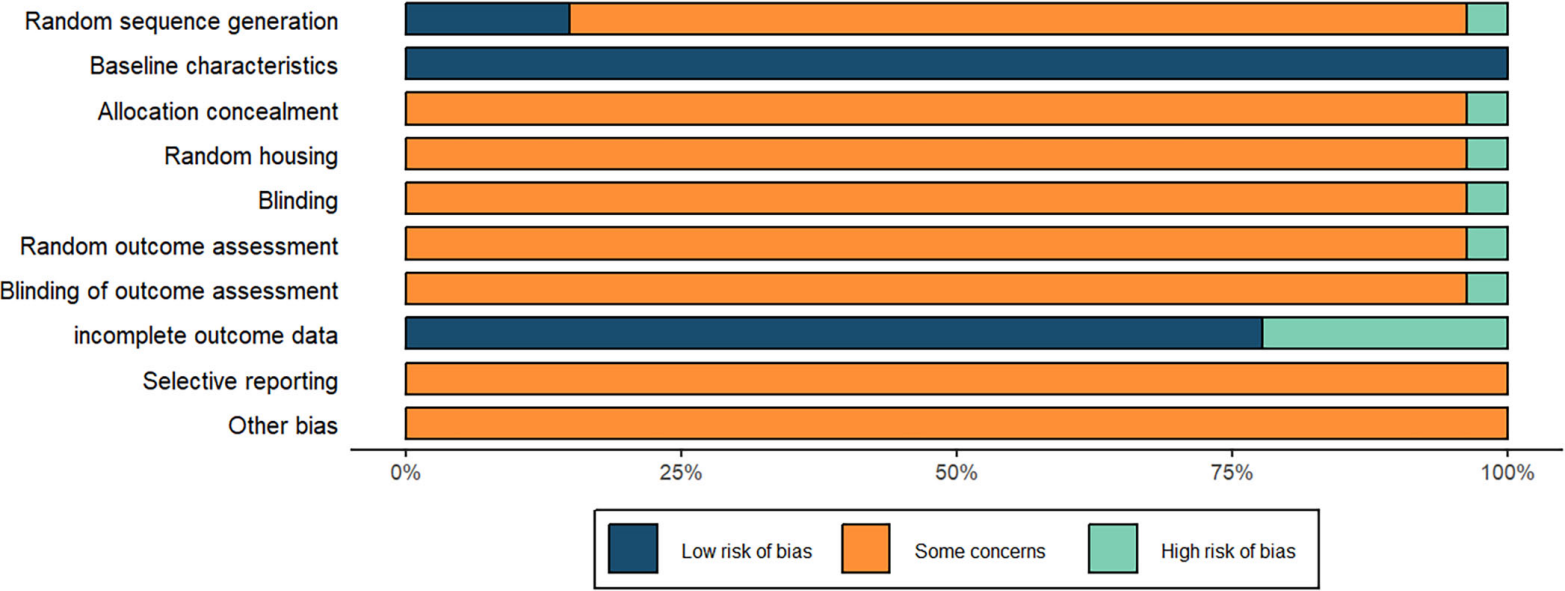
Risk of bias assessment.

This study was assessed using the SYRCLE Risk of Bias tool, and the results are presented in **Figure 2**. The experimental animals used in 27 studies were rats bred at the research institution under specific conditions and used exclusively to conduct the experiments, which ensured that the risk of baseline characteristics were all low; in terms of random sequence generation, four experiments were low risk (35, 41, 49, 53), all of which were assigned using a randomized number table for allocation, one experiment (31) was high risk because the study performed sequence generation based on the cage numbering rule, and the rest of the experiments did not specify the randomization method, so they were of unclear risk; as for allocation concealment, random housing, blinding, random outcome assessment and blinding of outcome assessment, only one experiment (31) was high risk because the study was based on cage numbering rules for sequence generation, which may have some degree of impact on allocation concealment, randomization of animal placement, and implementation of blinding, and the rest of the experiments were not explicitly stated, so they were unclear risks; in terms of incomplete outcome data, five experiments (31, 33, 36, 39, 44, 53) were high risk because portion of experimental animals died during the course of their experiments, and the rest of the experiments had no records of experimental deaths of animals and had complete data, so they were all low risk; and it was not clear about selective reporting of results and other sources of bias.

#### 3.1.4 Femoral BMD

**Figure 3:**
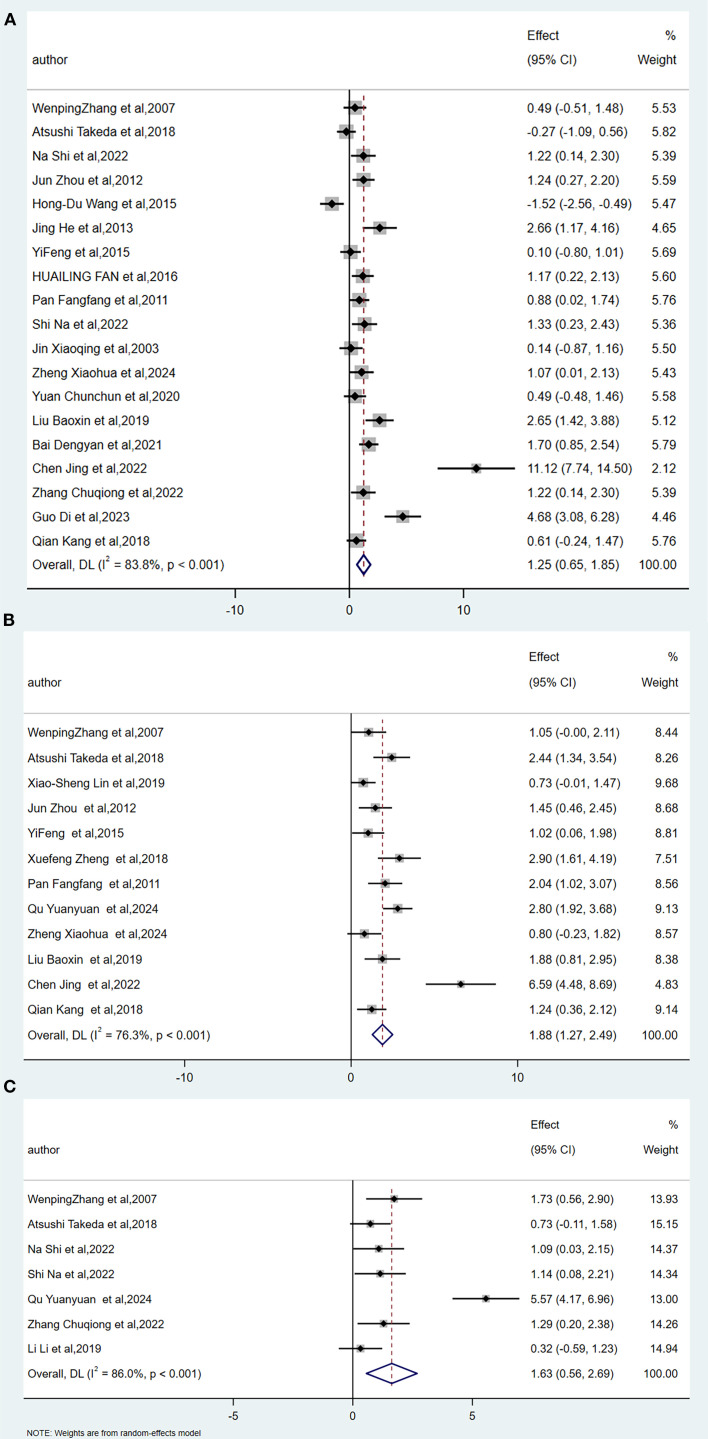
Forest plot of the correlation of acupuncture-related therapies with femoral BMD **(A)**, lumbar BMD **(B)**, and tibia BMD **(C)**.

A total of nineteen animal experiments (29–31, 33–37, 39, 40, 44, 46–51, 54, 55) were included in this meta-analysis, as shown in **Figure 3A**. Based on the heterogeneity results (I² = 83.8%, p < 0.001), significant heterogeneity was observed among the studies; therefore, a random-effects model was used for data analysis. The combined effect size was 1.25, with a 95% confidence interval of 0.65 to 1.87, and the difference was statistically significant. These results indicate that the acupuncture-related therapy group showed significantly greater improvement in femoral bone mineral density (BMD) compared to the conventional treatment group.

#### 3.1.5 Lumbar BMD

A total of nineteen animal experiments (29–31, 33–37, 39, 40, 44, 46–51, 54, 55) were included in this meta-analysis, as shown in **Figure 3B**. Based on the heterogeneity results (I² = 83.8%, p < 0.001), significant heterogeneity was observed among the studies; therefore, a random-effects model was used for data analysis. The combined effect size was 1.88, with a 95% confidence interval of 1.27 to 2.49, and the difference was statistically significant. These results indicate that the acupuncture-related therapy group showed significantly greater improvement in femoral bone mineral density (BMD) compared to the conventional treatment group.

#### 3.1.6 Tibial BMD

A total of seven animal experiments (29–31, 40, 45, 51, 53) were included in this meta-analysis, as shown in **Figure 3C**. Heterogeneity was observed across studies based on the heterogeneity results (I² = 86%, p < 0.001); therefore, a random-effects model was used for data analysis. The combined effect size was 1.63, with a 95% confidence interval of 0.56 to 2.69, and the difference was statistically significant. These results indicate that the acupuncture-related therapy group showed significantly greater improvement in tibia bone mineral density (BMD) compared to the conventional treatment group.

#### 3.1.7 Estradiol

**Figure 4:**
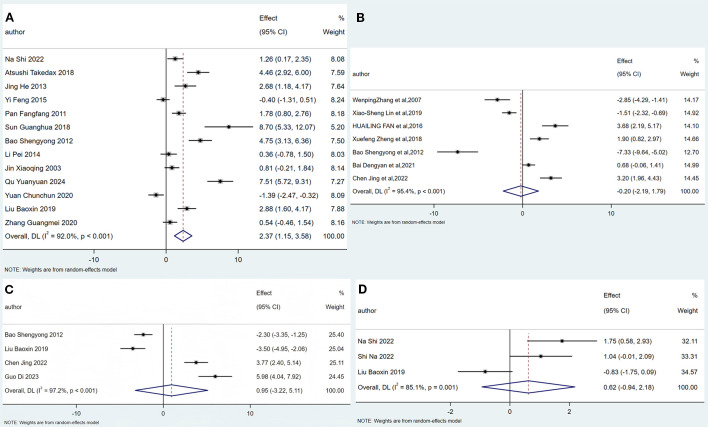
Forest plot of the correlation of acupuncture-related therapies with Estradiol **(A)**, Osteocalcin **(B)**, Alkaline Phosphatas **(C)** and Blood Calcium **(D)**.

A total of thirteen animal experiments (30, 31, 34, 36, 39, 41–48, 52) were included in this meta-analysis, as shown in **Figure 4A**. Based on the heterogeneity results (I² = 92%, P < 0.001), the studies were considered heterogeneous, and the data were analyzed using a random-effects model. The combined effect size was 2.37, with a 95% confidence interval of 1.15 to 3.58, and the difference was statistically significant. This indicates that the acupuncture-related therapy group performed significantly better than the conventional treatment group in improving E_2_ levels.

#### 3.1.8 Osteocalcin

A total of seven animal experiments (29, 32, 37, 38, 42, 49, 50) were included in this meta-analysis, as shown in **Figure 4B**. Significant heterogeneity was observed across studies (I² = 94.0%, p < 0.001), so data were analyzed using a random-effects model. The combined effect size was -0.20, with a 95% confidence interval of (-2.19, 1.79), and the difference was not statistically significant. These results indicate that the acupuncture-related therapy group was not significantly more effective than the conventional treatment group in improving osteocalcin levels.

#### 3.1.9 Alkaline phosphatase

A total of four animal experiments (42, 48, 50, 54) were included in this meta-analysis, as shown in **Figure 4C**. Significant heterogeneity was observed across studies (I² = 97.2%, p < 0.001), so the data were analyzed using a random-effects model. The combined effect size was 0.62, with a 95% confidence interval of -3.22 to 5.11, indicating that the difference was not statistically significant. This suggests that the acupuncture-related therapy group was not significantly more effective than the conventional treatment group in improving alkaline phosphatase levels.

#### 3.1.10 Blood calcium levels

A total of three animal experiments (30, 40, 48) were included in this meta-analysis, as shown in **Figure 4D**. The studies were considered heterogeneous based on the heterogeneity results (I² = 84.1%, p = 0.001), so the data were analyzed using a random-effects model. The combined effect size was 0.62, with a 95% confidence interval of (-0.94, 2.18), and the difference was not statistically significant. This indicates that the acupuncture-related therapy group was not significantly better than the conventional treatment group in improving blood calcium levels.

#### 3.1.11 Publication bias

**Figure 5:**
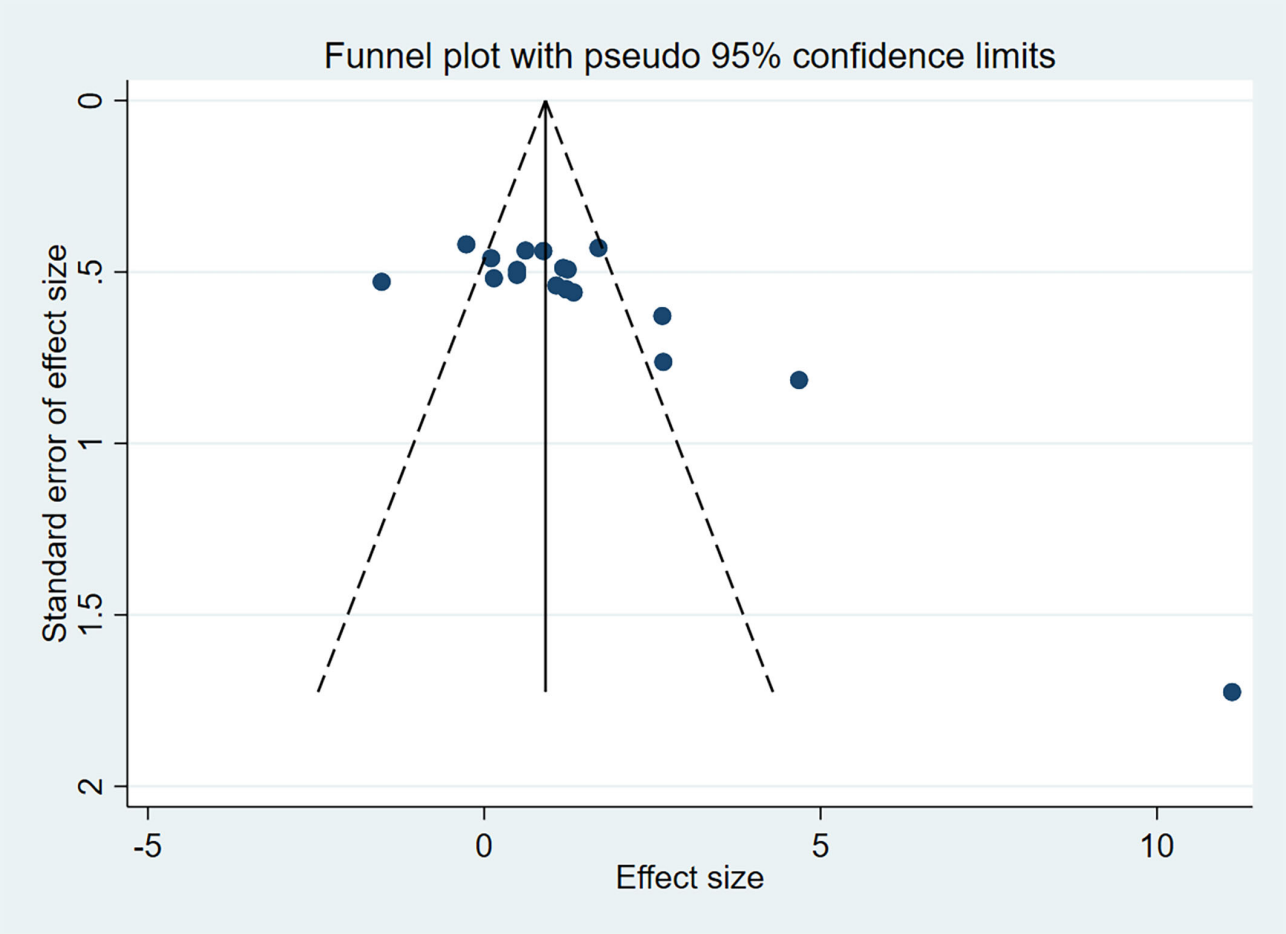
Funnel plot analysis to detect publication bias.

Since femoral BMD was the most frequently included indicator, it was tested for publication bias in this study, as shown in **Figure 5**. In the funnel plot, the distribution of scatter points is not entirely symmetrical, suggesting the presence of publication bias. Possible reasons for this bias include the small sample sizes of the included studies, low quality of some literature, and the non-publication of certain negative results.

### 3.2 Data mining

#### 3.2.1 Literature screening

A total of 27 relevant studies were included after systematic screening, involving 22 acupoints with a combined frequency of 77 occurrences.

#### 3.2.2 Frequency analysis

Frequency analysis was performed to obtain 9 common acupoints with a frequency greater than 10%, including Shenshu (BL23), Zusanli (ST36), Guanyuan (CV4), Sanyinjiao (SP6), Mingmen (GV4), Pishu (BL20), Xuanzhong (GB39), Huantiao (GB30), and Jingmen (GB25), as shown in **Table 2**.

**Table 2:** Common acupoints with a frequency greater than 10% (Located in rats).

Rank	Acupoint	Frequency (nl%)	Anatomical location
1	BL23	16(59.26%)	5mm lateral to the inferior part of the spinous process of the second lumbar vertebra.
2	ST36	14(51.85%)	below the lateral side of the knee joint of the hind limb, about 3–5 mm below the tibial tuberosity.
3	CV4	8(29.63%)	25 mm below the umbilicus,located on the linea alba.
4	SP6	6(22.22%)	3 - 5mm directly above the medial malleolus on the medial side of the hind - limb shin.
5	GV4	5(18.52%)	at the depression below the spinous process of the second lumbar vertebra.
6	BL20	4(14.81%)	3 - 5mm lateral to the inferior part of the spinous process of the 11th thoracic vertebra.
7	GB39	4(14.81%)	3–5 mm above the tip of the lateral malleolus of the hind limb.
8	GB30	3(11.11%)	The posterior - superior border of the hip joint of the hind limb.
9	GB25	3(11.11%)	Below the free end of the 12th rib.

#### 3.2.3 Association rule analysis

**Figure 6:**
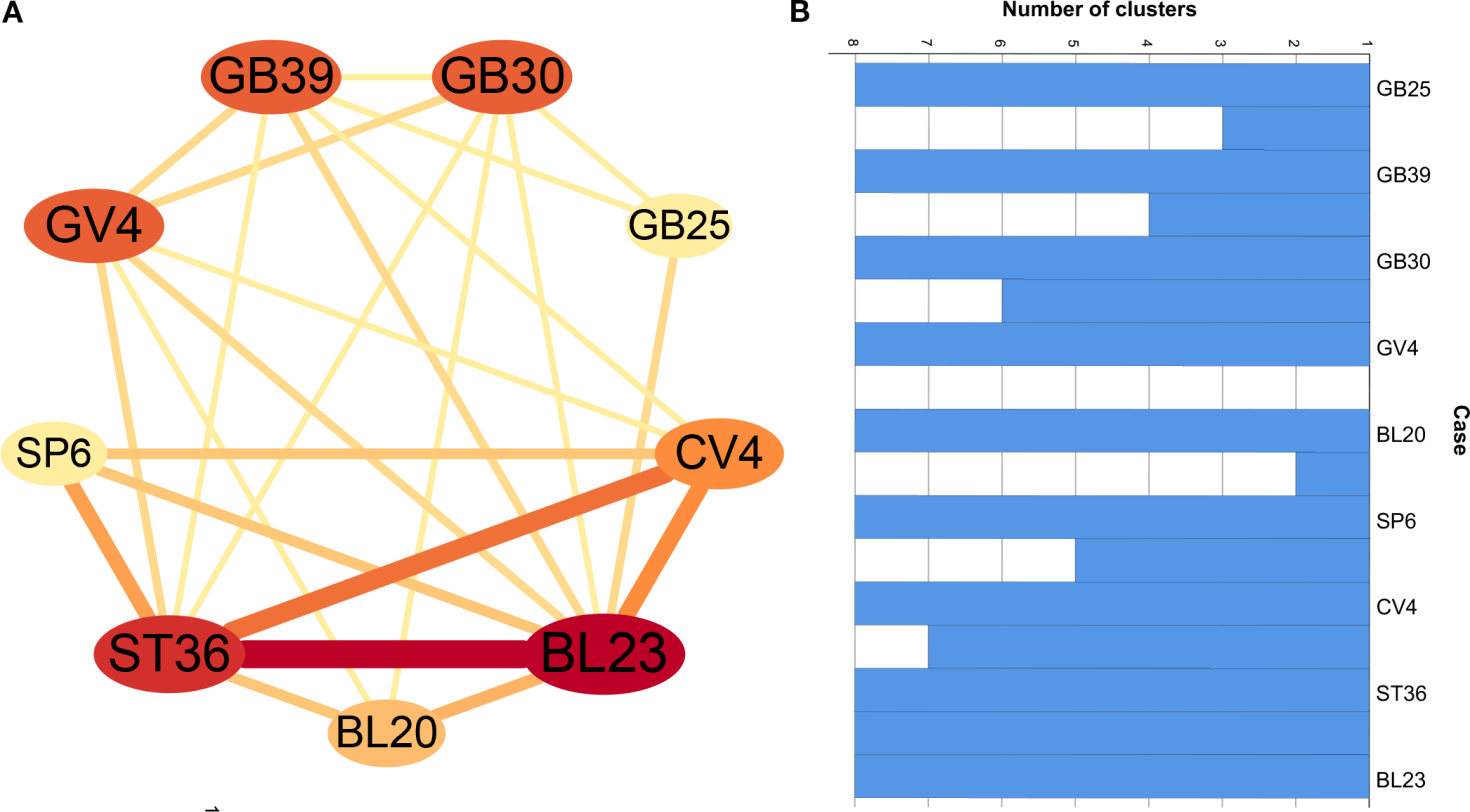
Network relationship for common acupoints **(A)** and icicle chart **(B)** of the cluster analysis of acupuncture-related therapies for POMP.

Association rule analysis was conducted to identify core acupoint combinations formed by commonly used acupoints. Under the conditions of support ≥ 20%, confidence ≥ 75%, and lift ≥ 1.0, with the antecedent items set to 2, six core acupoint combinations were identified, as shown in **Table 3**. These combinations involved Zusanli (ST36), Shenshu (BL23), Guanyuan (CV4), and Sanyinjiao (SP6), indicating that these acupoints are central to acupuncture treatment for PMOP. The network relationship diagram is presented in **Figure 6A**.

**Table 3:** Association rules analysis of common acupoints.

Acupoint combination	Frequency/n	Support (%)	Confidence (%)	Lift
ST36, BL23	14	51.85	78.57	1.33
CV4, ST36	8	29.63	87.50	1.69
CV4, BL23	8	29.63	75.00	1.27
CV4, ST36, BL23	7	25.93	85.71	1.45
SP6, ST36	6	22.22	83.33	1.61
CV4, BL23, ST36	6	22.22	100.00	1.93

#### 3.2.4 Cluster analysis

**Figure 7:**
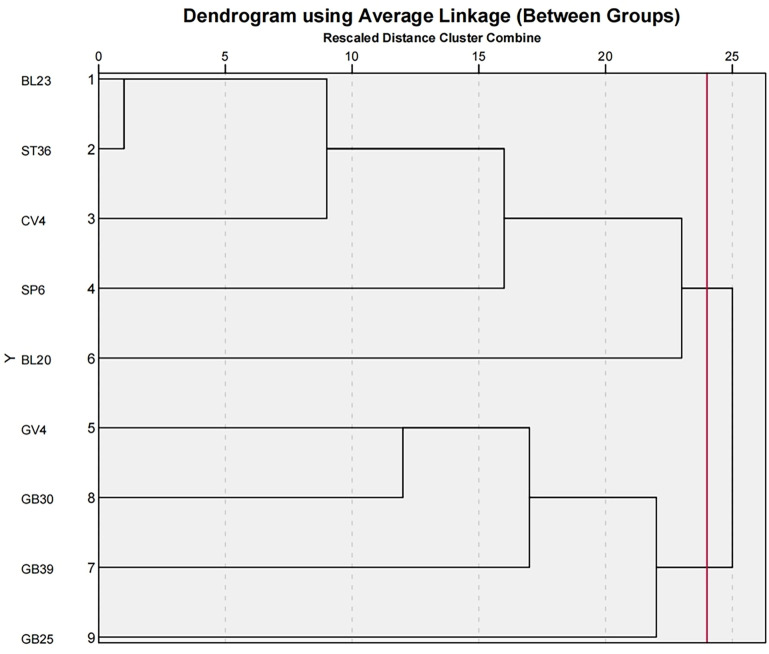
Tree chart of the cluster analysis of acupuncture-related therapies for POMP.

**Figure 8:**
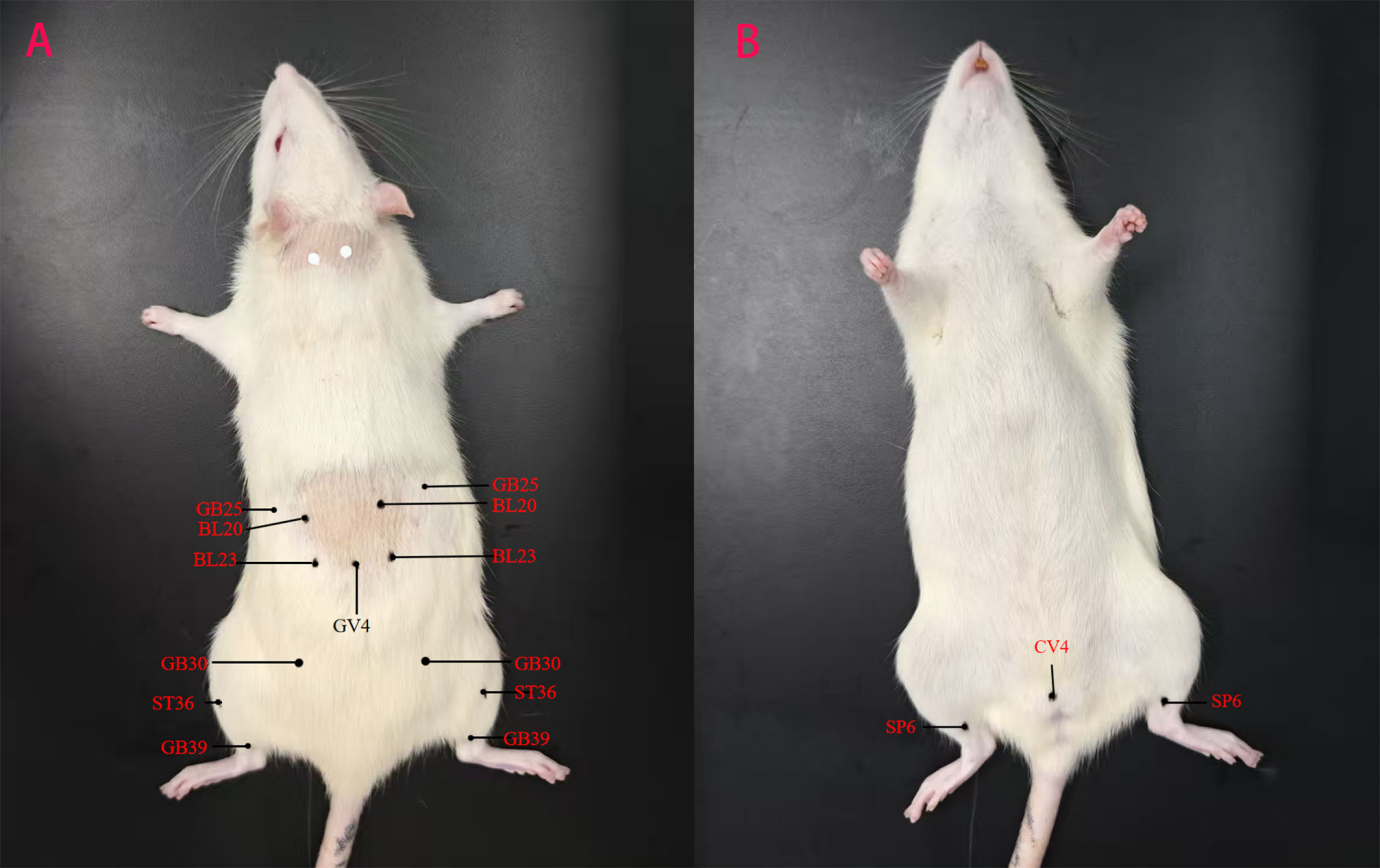
Data mining summarizes acupoints for treating postmenopausal osteoporosis in rats.

Cluster analysis, a statistical technique used to group similar items into distinct, relatively homogeneous categories, was applied to the top nine high-frequency acupoints (frequency ≥ 3). The Jaccard similarity coefficient measured acupoint similarity, and the average linkage method served as the agglomerative algorithm. The analysis was performed using SPSS Statistics 26.0 software. The results, visualized as an icicle chart (**Figure 6B**) and dendrograms (**Figure 7**), revealed two major clusters at a distance scale of 24.0: Cluster 1 included Shenshu (BL23), Zusanli (ST36), Guanyuan (CV4), Sanyinjiao (SP6), and Pishu (BL20); Cluster 2 comprised Mingmen (GV4), Xuanzhong (GB39), Huantiao (GB30), and Jingmen (GB25). The acupoints identified through data mining for the postmenopausal osteoporosis rat model are shown in **Figure 8**.

## 4 Discussion

### 4.1 Research background and significance

Postmenopausal osteoporosis is a metabolic bone disease characterized by decreased estrogen levels and reduced bone density in postmenopausal women (56). Current treatments primarily involve medication (57), which mainly work by inhibiting bone resorption through estrogen supplementation, thereby reducing bone mass loss and improving bone metabolism disorders. These interventions aim to treat or alleviate the symptoms of postmenopausal osteoporosis. However, existing mainstream therapies have limited ability to reverse bone loss or stimulate new bone formation. Additionally, these drugs are associated with side effects and risks, including cardiovascular disease, stroke, and breast cancer (58), which can reduce patient compliance and hinder the overall effectiveness of osteoporosis treatment.

Acupuncture has been reported to have unique efficacy and a favorable safety profile in treating postmenopausal osteoporosis. In 2003, Jin Xiaoqing (44) demonstrated that acupuncture therapy significantly improved bone mineral density and estradiol levels in PMOP rats. Additionally, further studies have confirmed that acupuncture-related therapies not only effectively increase bone mineral density in animal models of PMOP but also influence sympathetic nerve activity (29) and intestinal flora (30) to some extent in these models. These findings suggest that acupuncture-related therapies have the potential to treat PMOP and its complications.

To our knowledge, this is the first meta-analysis and data mining study of acupuncture-related therapies for postmenopausal osteoporosis (PMOP). The study aims to evaluate the benefits of these therapies and to investigate the acupoint selection strategies used in their treatment.

### 4.2 Evaluation of efficacy

This Meta-analysis included 27 animal studies with 548 animals.

The results of the meta-analysis demonstrated that acupuncture has a significant advantage in increasing estradiol (E_2_) levels compared to conventional controls. Recent studies (59) have shown that E_2_ regulates bone density by influencing estrogen receptors in bone and skeletal muscle. Consequently, postmenopausal osteoporosis frequently occurs in women after menopause, suggesting that increasing E_2_ levels may alleviate symptoms of this condition. Acupuncture-related therapies have already been proven effective in improving postmenopausal discomfort symptoms. For example, a 2011 clinical trial (60) demonstrated that acupuncture significantly reduces hot flashes in postmenopausal women. Furthermore, a 2016 systematic review by Hsiao-Yean Chiu et al. (61) confirmed the excellent therapeutic effects of acupuncture on sleep disorders in postmenopausal women. These findings clearly indicate that acupuncture-related therapies are clinically effective in treating postmenopausal disorders.

Previous research demonstrated that acupuncture-related therapies could improve clinical symptoms of postmenopausal-related diseases. However, it was not until 2021 that Chang Liu and colleagues (62) showed that acupuncture could significantly increase estradiol (E_2_) levels, specifically for treating postmenopausal conditions such as osteoporosis. The current study reviews existing animal experiments and further confirms that acupuncture-related therapies significantly elevate estradiol, a hormone associated with postmenopausal osteoporosis. Additionally, it supports the potential for investigating the physiological mechanisms of acupuncture in treating postmenopausal osteoporosis through animal models.

Regarding bone mineral density (BMD), the results of the meta-analysis demonstrated that acupuncture-related therapies were effective in enhancing bone density in experimental animals compared to routine treatments. In terms of anatomical location, the number of experiments involving the femur and lumbar spine included in this study exceeded ten. This is primarily because femoral neck fractures caused by osteoporosis, as well as lumbar vertebral compression fractures, are among the most serious complications of the disease (63), leading to a higher volume of related research. Notably, femoral fractures have been shown by Lingyu Lu et al. (64) in 2022 to respond significantly to pain relief in elderly patients. The physiological mechanism underlying the analgesic effect of acupuncture on the femoral region was elucidated in 2023 (65), demonstrating mediation through the recruitment of neutrophils and the release of β-endorphins. Regarding acupuncture-related therapies for lumbar compression fractures, relatively few studies exist. However, a clinical trial conducted in 2015 (66) confirmed the significant efficacy of acupuncture in alleviating pain and improving mobility disorders caused by lumbar compression fractures, with safety also established. Nevertheless, no studies have yet investigated the physiological mechanisms of acupuncture-related therapies for lumbar compression fractures.

Regarding OC, ALP, and blood calcium, the results of the meta-analysis showed that acupuncture-related therapies did not demonstrate a significant advantage in increasing OC, ALP, and blood calcium levels in experimental animals compared to the control group. However, individual animal studies indicated that acupuncture-related therapies could effectively enhance OC, ALP, and blood calcium levels. This suggests that while acupuncture-related therapies may have certain therapeutic effects, they do not offer significant advantages over current mainstream treatment regimens.

### 4.3 Data mining of acupuncture protocols

The frequency analysis identified nine common acupoints used in acupuncture for postmenopausal osteoporosis (PMOP). However, due to limitations in support and confidence levels, commonly used acupoints such as Dazhu (BL11) and Yanglingquan (GB34) were excluded from the association rule analysis. Although the analysis included frequently used acupoints for treating PMOP in the lower limbs, such as Xuanzhong (GB39) and Huantiao (GB30), these acupoints were either underutilized or lacked strong associations with other acupoints in the database constructed from the included studies. The association rule analysis revealed that the core acupoint combinations consisted of Zusanli (ST36), Shenshu (BL23), Guanyuan (CV4), and Sanyinjiao (SP6), indicating that these acupoints are central to acupuncture treatment for PMOP. The needling methods and location plans for these core acupoints are presented in **Table 2**.

Previous studies have demonstrated that moxibustion of Shenshu (BL23) and Zusanli (ST36) alleviates cartilage degradation and bone destruction, decreased the expression of RANKL mRNA while increased the expression of OPG mRNA, and intervented on expression of interleukin-1(IL-l) and tumor necrosis factor-alpha (TNF-alpha), to exhibite anti-inflammatory and anti-osteoporotic effects (67–69). Additional studies have shown that acupuncture at Zusanli (ST36) can induce expression in the hippocampus to exert analgesic effects and attenuates inflammation by suppressing M1 macrophage polarization (70). Furthermore, acupuncture at Shenshu (BL23) inhibits osteoclastogenesis, promotes bone formation, partially restores bone mineral density, and reverses osteoporosis progression, potentially through elevated plasma estradiol levels and reduced OPGL expression (71, 72). Another study revealed that both the subcutaneous abdominal adipose and the liver tissues contributed to the effects of electroacupuncture on the extragonadal aromatization,thus acupuncture at Guanyuan (CV4) can promote the blood concentrations of estrogen in the ovariectomized rats, thereby ameliorating PMOP (73).

According to traditional Chinese medicine theory, Zusanli (ST36) belongs to the stomach meridian, Shenshu (BL23) to the bladder meridian, Guanyuan (CV4) to the Conception Vessel, and Sanyinjiao (SP6) to the spleen meridian. Together, these acupoints play a role in invigorating the spleen and kidneys and replenishing the marrow, which corresponds to the pathogenic mechanism of PMOP with deficiency of kidney essence. In this study, cluster analysis categorized the core acupoints into two groups. The first group comprised Shenshu (BL23), Zusanli (ST36), Guanyuan (CV4), Sanyinjiao (SP6), and Pishu (BL20), primarily functioning to tonify the spleen and kidneys. The second group consisted of Mingmen (GV4), Xuanzhong (GB39), Huantiao (GB30), and Jingmen (GB25), primarily acting to fortify bones and regulate gallbladder function. These two sets of acupoints complement each other, collectively exerting therapeutic effects by tonifying the spleen and kidneys as well as fortifying bones and regulating gallbladder function, aligning with the Chinese medical pathogenesis of PMOP. Based on these results, we recommend Shenshu (BL23), Zusanli (ST36), Guanyuan (CV4), Sanyinjiao (SP6), Mingmen (GV4), Pishu (BL20), Xuanzhong (GB39), Huantiao (GB30), and Jingmen (GB25) as the selected acupoints for the treatment of PMOP.

In the treatment of postmenopausal osteoporosis (PMOP), the Bladder Meridian (BL) and Gallbladder Meridian (GB) are commonly utilized. As the Foot-Taiyang Meridian, the BL is externally and internally connected to the Kidney Meridian and contains all the Back-Shu points of the five Zang organs and six Fu organs. It is the meridian richest in yang qi in the body, responsible for regulating systemic yang qi and expelling pathogens. The GB, as the Foot-Shaoyang Meridian, governs pivotal activities and decision-making, is externally and internally linked to the Liver Meridian, and regulates the ascending, descending, entering, and exiting of qi.

The core pathogenesis of postmenopausal osteoporosis (PMOP) lies in kidney essence deficiency and marrow insufficiency, with the therapeutic principle focused on replenishing essence and nourishing the marrow. Shenshu (BL23), located on the Bladder meridian where kidney essence converges, can tonify the kidney, replenish essence, strengthen bones, and generate marrow. Pishu (BL20) can fortify the spleen, harmonize the stomach, and promote the production of qi and blood. Their combination significantly enhances the effects of replenishing essence and nourishing the marrow. Xuanzhong (GB39), the Confluence Point of Marrow on the Gallbladder meridian, is a key acupoint for gathering systemic essence, capable of nourishing bone marrow and strengthening tendons and bones. Yanglingquan (GB34), the Confluence Point of Tendons, can relax tendons, relieve spasms, and harmonize qi and blood. The combination of Xuanzhong (GB39) and Yanglingquan (GB34) promotes sufficient marrow, strengthens tendons, and alleviates osteoporosis.

Both the Bladder (BL) and Gallbladder (GB) meridians belong to the yang category. BL assists GB in promoting the ascension of yang qi, while GB aids BL in regulating qi movement. Their synergy enhances the effectiveness of replenishing essence and nourishing marrow. Additionally, patients with postmenopausal osteoporosis (PMOP) often exhibit emotional symptoms such as depression and anxiety. The GB meridian is particularly effective in regulating emotions; when combined with BL, it not only addresses the root cause by replenishing essence and nourishing marrow but also alleviates emotional symptoms, thereby achieving a holistic treatment of both body and spirit.

### 4.4 Mechanisms of acupuncture-related therapies for PMOP

The development and progression of postmenopausal osteoporosis are often associated with reduced osteoblast differentiation and increased osteoclast formation. The TNF-α/NF-κB signaling pathway, an inflammation-related pathway, plays a key role in osteoclastogenesis. Activation of this pathway promotes osteoclast formation, which can initiate or exacerbate postmenopausal osteoporosis. Recent studies have shown that modulating inflammatory factors can help regulate bone health (67).

Acupuncture-related therapies can play a significant role in regulating inflammatory factors, thereby providing therapeutic benefits for postmenopausal osteoporosis. A 2019 systematic review demonstrated that acupuncture can reduce inflammatory responses by enhancing vagal activity (68). Additionally, acupuncture has shown similar modulatory effects on inflammatory factors in animal models. Researchers found that changes in the microenvironment of acupoints represent the initial response to acupuncture stimulation. This initial response involves PROKR2 neurons, high-threshold fine nerve fibers, activation of the cannabinoid CB2 receptor (CB2R), and Ca2+ influx. Ultimately, the cholinergic anti-inflammatory pathway of the nervous system, the adrenal dopamine anti-inflammatory pathway, and the sympathetic nervous system play key roles in transmitting acupuncture signals and suppressing systemic inflammation (70).

Regarding the selection of acupoints, the data mining segment of this study identified Zusanli (ST36), Shenshu (BL23), Guanyuan (CV4), and Sanyinjiao (SP6) as the core acupoints for acupuncture-related therapies in postmenopausal osteoporosis (PMOP). Zusanli (ST36) has demonstrated significant anti-inflammatory and antioxidant effects through vagus nerve activation across various systems, organs, and body fluids (10). Shenshu (BL23) effectively mitigates oxidative stress damage (73). Guanyuan (CV4) modulates the inflammatory response and macrophage polarization in obese adipose tissue (74). All of these acupoints exhibit strong anti-inflammatory properties and serve as valuable therapeutic targets in the treatment of postmenopausal osteoporosis.

### 4.5 Limitations

However, there were several limitations to be taken into consideration. First, the animal experiments included in the study were not comprehensive enough in measuring postmenopausal osteoporosis-related indicators. For example, only one study (48) measured parathyroid hormone (PTH) among the studies included in the study. Secondly, the course of treatment included in the study was between 30 days and 24 weeks, so the meta-analysis results mainly reflected the short-term efficacy of acupuncture in treating PMOP. Finally, insufficient randomization concealment. Some animal experiments lacked a detailed description of their randomization concealment procedures, which can lead to selection bias.

## 5 Conclusion

In this study, we analyzed animal literatures that investigated how acupuncture-related therapies might ameliorate low bone mineral density in PMOP models.

Acupuncture-related therapies can significantly improve low BMD and low E_2_ levels associated with postmenopausal osteoporosis.

Zusanli (ST36), Shenshu (BL23), Guanyuan (CV4), and Sanyinjiao (SP6) are the core acupoints for Acupuncture-Related Therapies in PMOP, and this program is expected to become a supplementary treatment for PMOP.

## Data Availability

The original contributions presented in the study are included in the article/**Supplementary Material**. Further inquiries can be directed to the corresponding authors.
