# Supplementary material for: The effect of acupuncture-related therapies in animal model of postmenopausal osteoporosis: a meta-analysis and data mining approach
Source: Front Endocrinol (Lausanne). 2025 Oct 15;16:1617154. doi: 10.3389/fendo.2025.1617154 (PMC12568401; doi:10.3389/fendo.2025.1617154)
Supplement: Supplementary file 1 [file DataSheet1.docx]

The English search formula was:(((((((((((((((((((((((rat model) OR (mouse model)) OR (rat)) OR (mouse)) OR (animal model))) OR (mice)) OR (murine)) OR (rabbit) OR (rats laboratory)) OR (laboratory rat)) OR (laboratory rats)) AND ((((Post-Menopausal Osteoporosis) OR (postmenopausal osteoporosis)) OR (PMOP)))) AND ((((((((((((((((((((((((((((acupuncture) OR (electroacupuncture)) OR (warm needling)) OR (fire needling)) OR (pressing needling)) OR (transcutaneous electrical acupoint stimulation)) OR (acupoint catgut embedding)) OR (acupoint injection)) OR (Electric acupuncture)) OR (Electroacupuncture therapy)) OR (Electric acupuncture therapy)) OR (EA)) OR (moxibustion)) OR (catgut implantation at acupoint)) OR (acupoint sticking therapy)) OR (Point Embedding Therapy)) OR (Acupoint Tread-embedding)) OR (Pharmacopuncture)) OR (Warm needle)) OR (Blood-letting therapy)) OR (Acupoint injection)) OR (acupuncture-related therapy)) OR (otopoint sticking)) OR (ear acupuncture)) OR (Auricular acupuncture therapy)) OR (otopoint seed-pressing)) OR (auricular pressure)) OR (thread embedding therapy))).

The Chinese search formula is:("绝经后骨质疏松症" OR "绝经后骨质疏松" OR "绝经后骨量减少") AND("大鼠模型" OR "小鼠模型" OR "大鼠" OR "小鼠" OR "动物模型" OR "兔子" OR "实验大鼠" ) AND("针刺" OR "电针" OR "温针" OR "火针" OR "经皮穴位电刺激" OR "穴位埋线" OR "穴位注射" OR "穴位贴敷" OR "针灸" OR "针法" OR "温针灸" OR "艾灸" OR "放血疗法" OR "耳针" OR "电针疗法" OR "穴位埋线疗法" OR "针灸相关疗法" OR "耳穴压豆" OR "揿针" OR "埋针").
